# Towards a Next-Generation Sequencing Diagnostic Service for Tumour Genotyping: A Comparison of Panels and Platforms

**DOI:** 10.1155/2015/478017

**Published:** 2015-08-17

**Authors:** George J. Burghel, Carolyn D. Hurst, Christopher M. Watson, Phillip A. Chambers, Helen Dickinson, Paul Roberts, Margaret A. Knowles

**Affiliations:** ^1^Yorkshire Regional Genetics Service, St. James's University Hospital, Leeds LS9 7TF, UK; ^2^Leeds Institute of Cancer & Pathology, University of Leeds, St. James's University Hospital, Leeds LS9 7TF, UK; ^3^Leeds Institute of Biomedical & Clinical Sciences, University of Leeds, St. James's University Hospital, Leeds LS9 7TF, UK

## Abstract

Detection of clinically actionable mutations in diagnostic tumour specimens aids in the selection of targeted therapeutics. With an ever increasing number of clinically significant mutations identified, tumour genetic diagnostics is moving from single to multigene analysis. As it is still not feasible for routine diagnostic laboratories to perform sequencing of the entire cancer genome, our approach was to undertake targeted mutation detection. To optimise our diagnostic workflow, we evaluated three target enrichment strategies using two next-generation sequencing (NGS) platforms (Illumina MiSeq and Ion PGM). The target enrichment strategies were Fluidigm Access Array custom amplicon panel including 13 genes (MiSeq sequencing), the Oxford Gene Technologies (OGT) SureSeq Solid Tumour hybridisation panel including 60 genes (MiSeq sequencing), and an Ion AmpliSeq Cancer Hotspot Panel including 50 genes (Ion PGM sequencing). DNA extracted from formalin-fixed paraffin-embedded (FFPE) blocks of eight previously characterised cancer cell lines was tested using the three panels. Matching genomic DNA from fresh cultures of these cell lines was also tested using the custom Fluidigm panel and the OGT SureSeq Solid Tumour panel. Each panel allowed mutation detection of core cancer genes including *KRAS*, *BRAF*, and *EGFR*. Our results indicate that the panels enable accurate variant detection despite sequencing from FFPE DNA.

## 1. Introduction

Genetic factors play a principal role in cancer predisposition, initiation, and development [1–3]. Throughout cancer progression, the genome acquires somatic genetic and epigenetic changes [4, 5]. Some mutations play a critical role in cancer development by affecting key cancer “driver” genes. In contrast, some changes do not provide any selective advantage and are termed “passenger” mutations [6]. A number of the “driver” gene mutations are considered “actionable” as they have diagnostic, prognostic, or predictive implications. In addition, some of these mutations can be targeted by specific therapies and are commonly termed “druggable” variants [7].

Identification of “actionable” mutations informs clinical management and can be used to personalise cancer therapy. This is proving to be safer and more effective than traditional approaches [7, 8]. Many such genetic biomarkers are already in clinical use. Examples include* EGFR* mutation status as a predictive marker for sensitivity or resistance to anti-EGFR therapies including erlotinib/gefitinib in non-small cell lung cancer (NSCLC) and* BRAF* mutation status as a predictive marker for the B-Raf inhibitor, Vemurafenib, associated with metastatic melanoma [7, 9–11]. Different molecular techniques are in use in diagnostic laboratories to assess oncogenic genetic biomarkers, including fluorescence* in situ* hybridisation (FISH), quantitative polymerase chain reaction (Q-PCR) (for amplifications or rearrangements), and Sanger sequencing and pyrosequencing (for point mutations and small insertions or deletions). These techniques are frequently labour intensive, require large amounts of DNA, and are relatively slow and expensive. They often have a high failure rate and a limited scalability to expand analyses to additional genes.

As more clinically significant genetic biomarkers and targeted therapies become available, diagnostic testing of these genes has become more challenging. In addition to the larger number of targets, challenges include poor quality formalin-fixed paraffin-embedded (FFPE) DNA samples, small sample size (e.g., needle biopsies), tumour sample heterogeneity, and turn-around times [11, 12]. The introduction of high throughput and lower cost sequencing technologies, especially next-generation sequencing (NGS), has revolutionised cancer genomics research and diagnostics [7] which are rapidly moving from single gene mutation analysis to cancer genome profiling [8, 13]. Nevertheless, sequencing of the entire cancer genome by routine diagnostic laboratories is currently unfeasible as it remains expensive, time consuming, and labour intensive. Therefore, approaches to targeted resequencing are necessary to capture and enrich specific genomic regions of interest (cancer gene panels) from DNA samples prior to sequencing [14]. Several cancer gene panels and enrichment methods have been developed recently and validated for clinical diagnostic use by both research laboratories and commercial companies [8, 11, 12, 15, 16]. In the Yorkshire Regional Genetics Service (YRGS), Illumina NGS technology is routinely used to identify germline mutations causing hereditary cancer [17, 18]. Recently, a manual PCR-based enrichment method followed by NGS was developed and is currently used to sequence actionable somatic mutations in four key oncogenes:* BRAF*,* EGFR*,* KRAS*, and* PIK3CA* [13]. However, the manual nature of the enrichment method makes it technically challenging to expand the panel to incorporate additional cancer genes.

In order to streamline and enhance the YRGS diagnostic workflow, we evaluated three target enrichment strategies using two NGS platforms (Illumina MiSeq and Ion PGM). The target enrichment strategies were a Fluidigm Access Array custom amplicon panel including 13 genes (sequenced on the MiSeq), the Oxford Gene Technologies (OGT) SureSeq Solid Tumour hybridisation panel including 60 genes (sequenced on the MiSeq), and an Ion AmpliSeq Cancer Hotspot Panel including 50 genes (sequenced on the IonPGM). Eight previously characterised cancer cell lines were used to evaluate these approaches. DNA samples extracted from FFPE blocks of these cell lines were tested using the three panels. Matching genomic DNA samples extracted from fresh cultures of these cell lines were also tested using the custom Fluidigm panel and the OGT SureSeq Solid Tumour panel.

## 2. Materials and Methods

### 2.1. Samples

Eight previously characterised human bladder cancer cell lines were used: 639V, 92-1, 97-7, J82, JO'N, KU-19-19, LUCC3, and UM-UC3. Cell line identity was confirmed using the Powerplex 16HS system (Promega, Southampton, UK) [19]. The 8 cell lines contained 22 known mutations in 6 genes:* BRAF*,* FGFR3*,* KRAS*,* NRAS*,* PIK3CA*, and* TP53* (Table 1). Supplementary Table 1, in Supplementary Material available online at http://dx.doi.org/10.1155/2015/478017, provides further details on the mutation status of these cell lines and the methods used in their previous molecular characterisation.

### 2.2. Cell Culture and Fixation

Cells were cultured in suitable culture medium supplemented with 10% foetal calf serum at 37°C with 5% CO_2_. Harvested cells from confluent cultures were washed with phosphate buffered saline and resuspended in CytoRich red fixative (Becton Dickinson, Oxford, UK) before transport to the Leeds Cytopathology Department. The cells were then pelleted and fixed following standard operating procedures used to produce cell blocks from clinical samples such as fine needle aspirates.

### 2.3. DNA Extraction

Genomic DNA (gDNA) from cultured cells was extracted using a QIAamp DNA mini kit (Qiagen, Manchester, UK) according to the manufacturer's instructions. DNA was extracted from FFPE sections using a BioRobot EZ1 automated extractor (Qiagen, Manchester, UK) according to the manufacturer's instructions.

### 2.4. Target Enrichment

The design of Fluidigm enrichment primers was undertaken by the Fluidigm design team with a target size range of 150–160 bp. Target enrichment was performed according to the manufacturer's instructions using the Access Array System for Illumina Sequencing Systems v.G1 (Fluidigm UK Limited, London, UK). The SureSeq hybridisation enrichment was performed according to the manufacturer's instructions (OGT) and with the support of the OGT technical team. The Ion AmpliSeq enrichment (Life Technologies Ltd., Paisley, UK) was performed according to the manufacturer's instructions. Supplementary Tables 2–4 list the targeted genes/exons in the three panels.

### 2.5. Sequencing

The Fluidigm custom panel and the SureSeq panel libraries were sequenced on the Illumina MiSeq (Fresh and FFPE gDNA were sequenced separately for each of the panels), which was carried out in the Translational Genomics Unit at St. James's University Hospital. This used Illumina MiSeq Reagent kit v2 (MS-102-2002) 300 cycles, Illumina Experiment Manager v1.6.0 for building library plates and creating the sample sheet, and the MiSeq Control Software (MCS) v2.3.0.8 for monitoring the runs and quality control. The Ion AmpliSeq panel libraries were sequenced on an Ion PGM, which was carried out by Life Technologies team in Paisley.

### 2.6. Bioinformatics Analysis

Sequence alignment and variant calling for the Fluidigm custom panel was performed using NextGene software v.2.3.4 (SoftGenetics LLC, State College, PA, USA) using defined parameters that included a minimum read depth of 500x and nonreference allele frequency of at least 5%. Sequence variants were visualised using the NextGene Viewer v.2.3.4. For the SureSeq panel, analysis was performed using the SureSeq Virtual Machine (SSVM) v.0.3, and, for the Ion AmpliSeq panel, Ion Reporter software v.4.0 was used. The data was filtered for variants in the 6 genes of interest and resulting variant calls were compared between the three panels. Novel sequence variants identified in regions previously investigated were confirmed using either bidirectional Sanger sequencing or pyrosequencing.

### 2.7. Sanger Sequencing

PCR products were enzymatically purified to remove residual primers and dNTPs using Illustra ExoProStar (GE Lifesciences) following the manufacturer's instructions. All sequencing reactions were carried out using the BigDye Terminator v.1.1 Cycle Sequencing Kit and were performed on a 3730 Genetic Analyzer (Life Technologies Ltd., Paisley, UK) according to the manufacturer's instructions. The sequencing data was analysed with reference to a control sequence trace using Mutation Surveyor software v.3.20 (SoftGenetics LLC, State College, PA, USA).

### 2.8. Pyrosequencing

Assays were performed by the Leeds Cytogenetics Laboratory using the Pyromark Q96 ID instrument (Qiagen, Manchester, UK) according to manufacturer's instructions. The pyrograms were analysed using Pyrogram ID v.2.5 (Qiagen, Manchester, UK).

## 3. Results

### 3.1. Next-Generation Sequencing

MiSeq cluster densities of 811 K/mm^2^ and 880 K/mm^2^ were achieved for the Fluidigm custom panel libraries prepared using fresh gDNA and FFPE gDNA, respectively. This corresponded to 15.21 and 16.62 million reads of which 90.63% and 35.37% passed filter, respectively. The fresh gDNA prepared pool yielded 2.1 Gb of data while the FFPE gDNA prepared pool yielded 0.8 Gb. The percentage of bases that were ≥Q30 for each pool was 94.7% (fresh gDNA) and 72.2% (FFPE gDNA). The per-sample read distribution is displayed in Supplementary Table 5 which indicates that the pooling was performed accurately.

MiSeq cluster densities of 788 K/mm^2^ and 767 K/mm^2^ were achieved for SureSeq Solid Tumour panel using fresh gDNA and FFPE gDNA, respectively. This corresponded to 16.53 million and 16.21 million reads of which 72.58% and 73.31% passed filter, respectively. Both SureSeq Solid Tumour panel pools yielded 1.8 Gb of data and the percentage of bases that were ≥Q30 per pool was approximately 88%. The per-sample read distribution is displayed in Supplementary Table 6, which indicates that the pooling was performed accurately.

The Ion PGM sequencing output information is shown in Supplementary Figure S1 as supplied by Life Technologies. The per-sample read distribution is displayed in Supplementary Table 7, which indicates that the pooling was performed accurately.

### 3.2. Analysis of Fresh Genomic DNA Samples

Using the Fluidigm custom panel, 20 of the 22 known mutations were correctly identified. The two undetected mutations were c.742C>T and c.746C>G in* FGFR3* exon 7, which were missed due to PCR failure. Two previously unknown mutations,* KRAS* c.38G>A and* TP53* c.783-2A>G, were identified in cell lines 92-1 and LUCC3, respectively. These were confirmed using Sanger sequencing (*TP53* c.783-2A>G) and pyrosequencing (*KRAS* c.38G>A).

**Figure 1:**
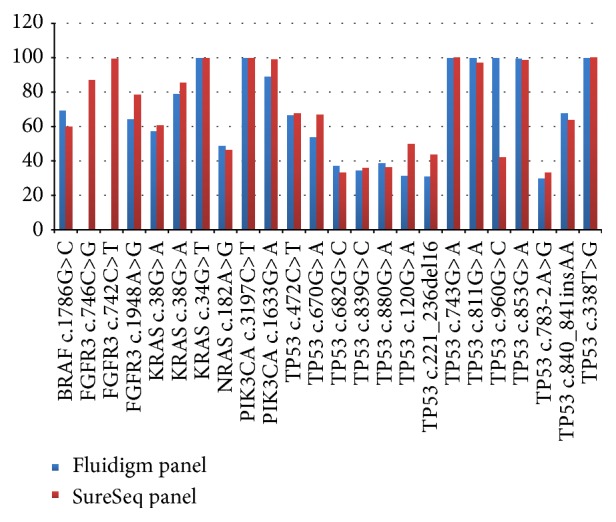
Mutant allele frequency detected using fresh genomic DNA. Comparison of mutant allele frequencies detected by the Fluidigm panel and the SureSeq panel.

Using the SureSeq panel, all of the known mutations were detected and the two novel mutations identified by the Fluidigm custom panel were confirmed. The depth of coverage and mutant allele frequency of all of the variants are summarised in Supplementary Table 8.  Figure 1 summarises the mutant allele frequency of the 24 mutations using the Fluidigm panel and the SureSeq panel. For 21 of 22 mutations detected successfully by both platforms, the mutant allele frequency was comparable. The* TP53* mutation c.960G>C had a significantly different mutant allele frequency between the 2 platforms: 42.1% (SureSeq panel) and 99.7% (Fluidigm panel). Based on Sanger sequencing, the mutation appeared to be heterozygous (~50% mutant allele frequency). The depth of coverage and mutant allele frequency of the variants as detected by the Fluidigm and the SureSeq panels in fresh gDNA are summarised in Supplementary Table 8. Notably, three further variants (false positives) were identified in the SureSeq panel with <10% variant allele frequency (Table 2).

### 3.3. Analysis of FFPE Genomic DNA Samples

Using the Fluidigm panel, 20 of the 24 mutations were successfully detected. In addition to the* FGFR3* exon 7 c.742C>T and c.746C>G mutations, two other mutations in* TP53* exon 4 (c.120G>A) and intron 7 (c.783-2A>G) were not detected. These* TP53* regions failed to amplify from most of the FFPE DNA samples. Using the SureSeq panel, all of the mutations were successfully detected.

**Figure 2:**
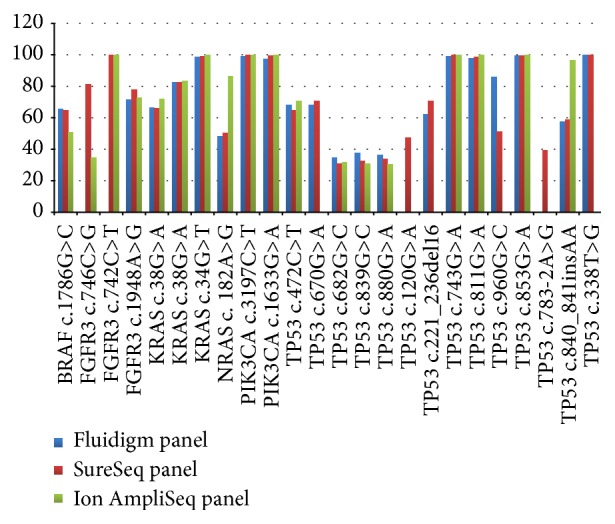
Mutant allele frequencies detected in genomic DNA extracted from FFPE samples. Comparison of mutant allele frequencies detected by the Fluidigm panel, the SureSeq panel, and the Ion AmpliSeq panel.

Using the Ion AmpliSeq panel, 18 of the 24 mutations were detected. The 6 missed mutations were in* TP53* (c.120G>A, c.221_236del16, c.338T>G, c.670G>A, c.783-2A>G, and c.960G>C), which are not among the hotspot mutations detected by this panel. The depth of coverage and mutant allele frequency of all of the variants are summarised in Supplementary Table 9.  Figure 2 summarises the mutant allele frequency of the 24 mutations using the 3 panels.

The Fluidigm panel and the SureSeq panel had reproducible mutant allele frequencies between fresh gDNA and FFPE gDNA. There were four mutations with significant differences in mutant allele frequency between the three panels (Table 3). Based on Sanger sequencing, all were heterozygous (50%).

Notably, three further variants (false positives) were identified in the SureSeq panel with <10% variant allele frequency (Table 4). One of these,* TP53* c.1129A>C, had also been identified at low variant allele frequency in fresh gDNA.

## 4. Discussion

Over the past few years, cancer treatment has entered a new era in which diagnostic testing of clinically actionable genetic markers allows selection of appropriate targeted therapies, commonly referred to as personalised or stratified medicine [11]. We aimed to evaluate several NGS-based tumour genotyping diagnostic panels to integrate and expand the current diagnostic tumour genotyping assays performed by the YRGS.

The target enrichment strategies were a custom Fluidigm panel and 2 commercially available panels, the SureSeq Solid Tumour hybridisation panel and the Ion AmpliSeq Cancer Hotspot Panel.

The comparison between the three enrichment strategies was performed on eight cancer cell lines in which 22 mutations had been previously identified using either Sanger sequencing, single strand conformation polymorphism (SSCP) analysis, high-resolution melting (HRM) analysis, or a SNaPshot Multiplex System (Life Technologies Ltd., Paisley, UK). The NGS-based approaches identified two mutations that were previously not detected using conventional techniques. These results demonstrate the increased sensitivity of using NGS in comparison to other existing techniques.

All three platforms were able to detect at least 75% of the 24 mutations in the FFPE DNA samples: SureSeq panel (100%), Fluidigm panel (83%), and Ion AmpliSeq panel (75%). For the Fluidigm panel, one of the mutations not detected was in* FGFR3* exon 7. This amplicon had failed at the design stage. The other three mutations were detected in fresh gDNA but not in FFPE. These mutations were in regions that underperformed in FFPE gDNA compared to fresh gDNA. It is well established that FFPE tumour DNA samples are challenging to amplify and sequence due to damage resulting from the fixation process and due to sample heterogeneity. This is likely to be one of the sensitivity differences between fresh and FFPE gDNA samples. However, some optimisations may enhance the performance of the assay with FFPE samples. These include redesign of some of the target regions, particularly those that frequently failed to amplify. Some of these regions had lower read depth in comparison to other targets using the fresh gDNA samples, indicating that they were intrinsically more difficult to amplify. Moreover, examination of the Agilent Bioanalyser traces for the 44 FFPE gDNA libraries indicated that the tagging reaction was not as efficient for the FFPE gDNA samples as demonstrated by an abundance of unincorporated oligonucleotide adaptor sequences. Optimisation of the tagging protocol is likely to enhance the read depth of some of the target regions which may help to increase sensitivity and specificity.

For the Ion AmpliSeq panel, all of the “missed” mutations were not included in the hotspot mutations covered by this panel. Therefore, the analytical sensitivity of the panel was 100%. Nevertheless, the clinical sensitivity may be reduced due to the restricted nature of the hotspots panel and so less common mutations may be missed. This is particularly the case for tumour suppressor genes such as* TP53*, where inactivating mutations may occur throughout the gene. For such genes, coverage of all coding sequence is ideal.

The SureSeq panel sensitivity was 100%. However, average read depth was significantly lower than the PCR-based Fluidigm panel and the Ion AmpliSeq panel (Supplementary Tables 5 and 6). Moreover, several false positives variants were identified within the* TP53* gene. The OGT R&D and bioinformatics teams followed up these variants. Following detailed inspection, they concluded that these discrepancies were independent of the hybridisation assay and that they were an artefact of the bioinformatics pipeline. As the genotyping is independent of the capture assay, in order to resolve these issues, additional user refinement of base and mapping quality score thresholds may be required.

## 5. Conclusions

The three platforms tested all showed acceptable performance. However, custom panels with a smaller number of clinically relevant and actionable targets and genes are likely to be more suitable for diagnostic laboratories. Custom designs and bioinformatics pipelines addressing both mutation hotspots and the entire coding sequence of clinically relevant genes will be required. Such custom panels will likely reduce the cost of the assay and improve the depth of coverage, which may help to reduce false positive variant calls. Cost, DNA requirements, and turn-around times will also be critical in choosing a platform for the diagnostic service.

## Supplementary Material

Supplementary material table 1 provides the details of the 8 cell lines mutation status, supplementary material tables 2-4 list the target regions/genes included within the three different target enrichment panels; Fluidigm Access Array custom amplicon panel, the Ion AmpliSeq Cancer Hotspot Panel and the OGT SureSeq Solid Tumour hybridisation panel. Supplementary tables 5-7 provide the per-sample read distribution of the three different target enrichment panels. Supplementary table 8 shows the depth of coverage and mutant allele frequency of all of the variants detected in the cell lines' fresh gDNA samples using the Fluidigm custom panel and the OGT SureSeq panel. Supplementary table 9 shows the depth of coverage and mutant allele frequency of all of the variants detected in the cell lines' FFPE gDNA samples using the three target enrichment panels. Supplementary figure S1 shows the Ion PGM sequencing output information.

## Figures and Tables

**Table 1:** Mutation status of the 8 bladder cancer cell lines.

Cell line	*BRAF *(NM_004333.4)	*FGFR3 *(NM_00142.4)	*KRAS *(NM_033360.2)	*NRAS *(NM_002524.3)	*PIK3CA *(NM_006218.2)	*TP53 *(NM_000546.5)
639V		c.742C>T			c.3197C>T	c.743G>A

92-1						c.472C>T, c.670G>A, c.682G>C, c.839G>C, c.880G>A

97-7		c.746C>G				c.120G>A, c.221_236del16

J82		c.1948A>G				c.811G>A, c.960G>C

JO'N			c.38G>A			c.853G>A

KU-19-19				c.182A>G		

LUCC3	c.1786G>C				c.1633G>A	c.840_841insAA

UM-UC3			c.34G>T			c.338T>G

**Table 2:** Additional mutations identified using the SureSeq Solid Tumour panel in fresh gDNA samples.

Cell line	Gene	Variant	Variant allele frequency	Read depth
97-7	*TP53 *(NM_000546.5)	c.853G>A	6.22%	225
639V		c.1129A>C	6.48%	355
KU-19-19		c.1129A>C	7.61%	197

**Table 3:** Four mutations with significant differences in mutant allele frequency between the three panels.

Cell line	Gene	Transcript	Variant	Fluidigm custom panel	SureSeq Solid Tumour panel	Ion AmpliSeq Cancer Hotspot Panel	Sanger sequencing zygosity status
97-7	*FGFR3*	NM_00142.4	c.746C>G	N/A	81.4%	34.6%	Heterozygous
KU-19-19	*NRAS*	NM_002524.3	c.182A>G	48.2%	50.5%	86.3%	Heterozygous
J82	*TP53*	NM_000546.5	c.960G>C	85.7%	51.3%	N/A	Heterozygous
LUCC3	*TP53*	NM_000546.5	c.840_841insAA	57.7%	58.9%	96.4%	Heterozygous

**Table 4:** Extra mutations identified using the SureSeq Solid Tumour panel in FFPE gDNA.

Cell line	Gene	Variant	Variant allele frequency	Read depth
639V	*TP53 *(NM_000546.5)	c.113C>A	5.10%	294
KU-19-19		c.1129A>C	6.64%	271
LUCC3		c.1129A>C	5.10%	255
