# Supplementary figures and images for: Towards a Next-Generation Sequencing Diagnostic Service for Tumour Genotyping: A Comparison of Panels and Platforms
Source: Biomed Res Int. 2015 Aug 17;2015:478017. doi: 10.1155/2015/478017 (PMC4553307; doi:10.1155/2015/478017)

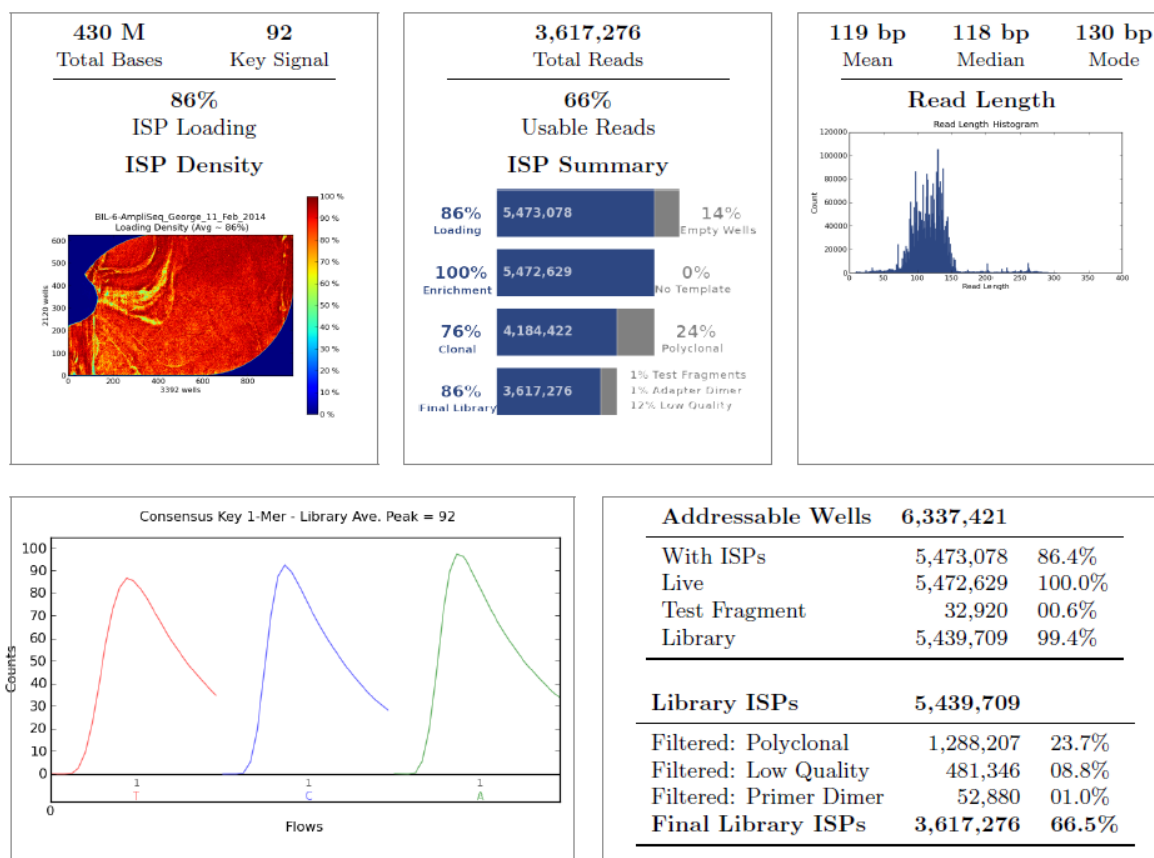

**Figure S1. Ion PGM Run Summary**

Supplement: Supplementary file 1 — Supplementary material table 1 provides the details of the 8 cell lines mutation status, supplementary material tables 2-4 list the target regions/genes included within the three different target enrichment panels; Fluidigm Access Array custom amplicon panel, the Ion AmpliSeq Cancer Hotspot Panel and the OGT SureSeq Solid Tumour hybridisation panel. Supplementary tables 5-7 provide the per-sample read distribution of the three different target enrichment panels. Supplementary table 8 shows the depth of coverage and mutant allele frequency of all of the variants detected in the cell lines' fresh gDNA samples using the Fluidigm custom panel and the OGT SureSeq panel. Supplementary table 9 shows the depth of coverage and mutant allele frequency of all of the variants detected in the cell lines' FFPE gDNA samples using the three target enrichment panels. Supplementary figure S1 shows the Ion PGM sequencing output information. [file 478017.f1.zip › 478017.f1/478017.v1.pdf]
